# Efficacy and safety of dexmedetomidine versus midazolam for sedation in mechanically ventilated pediatric patients: a randomized clinical trial

**DOI:** 10.1186/s44158-026-00370-2

**Published:** 2026-03-04

**Authors:** Majid Sezavar, Hosein Akhavan, Gholamreza Khademi, Maryam Naseri, Tahereh Sadeghi, Atiyeh Kaveh

**Affiliations:** 1 Department of Pediatrics, School of Medicine, Mashhad University of Medical Sciences, Mashhad, Iran; 2 Department of Pediatrics, School of Medicine, Sheikh Hospital, Mashhad University of Medical Sciences, Mashhad, Iran; 3 Department of Pediatrics, School of Medicine, Neonatal Research Center, Akbar Hospital, Mashhad University of Medical Sciences, Mashhad, Iran; 4 Department of Pediatrics, School of Nursing and Midwifery, Nursing and Midwifery Care Research Center, Mashhad University of Medical Sciences, Mashhad, Iran; 5 Department of Pediatrics, School of Medicine, Akbar Hospital, Mashhad University of Medical Sciences, Mashhad, Iran

**Keywords:** Dexmedetomidine, Midazolam, Sedation, Mechanical ventilation, PICU

## Abstract

**Aims:**

The benefits of Dexmedetomidne in sedation of mechanically ventilated pediatric patients are still challenging. In this study, we compared the efficacy and safety of Dexmedetomidine versus Midazolam for achieving deep sedation in pediatric patients receiving mechanical ventilation.

**Methods:**

A randomized clinical trial was conducted across three affiliated hospitals during February 2023 until February 2024. Eligible mechanically ventilated children aged 1 month to 18 years were randomized 1:1 to receive either Dexmedetomidine (0.25–1 mcg/kg/h) or midazolam (1–4 mcg/kg/min) for sedation, alongside fentanyl analgesia. Sedation was assessed using the Ramsey Sedation Scale and target scores set on 5–6. Primary outcomes included success rates in achieving target sedation at the beginning of mechanical ventilation; secondary outcomes encompassed hemodynamic adverse effects (hypotension, bradycardia), follow-up assessment of sedation level of successful patients over the next 6 h, mortality rate, mean arterial blood pressures and heart rates (HR) changes, mechanical ventilation and PICU stay duration.

**Main results:**

Among 130 eligible patients, 120 were analyzed after exclusions (60 per group). Demographic characteristics were similar between groups. The primary outcome revealed that Dexmedetomidine was less effective than Midazolam in achieving target sedation levels across all ages (p < 0.001). In infants under one year, there was no significant difference in sedation effectiveness between the two medications (P = 0.157). Notably, Dexmedetomidine patients required higher drug doses to maintain sedation over 6 h, while Midazolam patients experienced fewer adjustments (p < 0.001). Hemodynamic complications were significantly less frequent in the Dexmedetomidine group (3.3% vs. 23.3% for hypotension; p < 0.01). However, the two groups did not differ in terms of MAP and HR changes (P = 0.255; P = 0.063). Mortality, mechanical ventilation duration and length of PICU stay were not different between two groups (P = 0.853; P = 0.076; P = 0.082).

**Conclusion:**

Dexmedetomidine is inferior to Midazolam for deep sedation during mechanical ventilation in children aged 1 month to 18 years. However, it can be effective in infants under one year of age. Dexmedetomidine presents a lower risk of hemodynamic side effects compared to Midazolam.

**Trial registration:**

IRCT20190522043672N3 on February 16, 2023.

## Introduction

Critically ill children on mechanical ventilation often require analgesics and sedatives to alleviate pain and anxiety, improving comfort while minimizing patient-ventilator asynchrony and tracheal tube displacement [1]. Effective pain management in this population demands regular assessments and individualized treatment approaches The choice of medication is crucial for achieving optimal therapeutic outcomes while minimizing adverse effects [2].

Therapeutic sedation typically involves a combination of analgesics, such as morphine or fentanyl, and sedatives like benzodiazepines or Dexmedetomidine at the minimum effective doses [3]. Opioids provide pain relief via opioid receptors, while benzodiazepines enhance GABAergic activity to reduce anxiety and induce sedation [4, 5]. Midazolam is commonly used due to its rapid onset and short duration of action, making it suitable for procedures requiring quick recovery [6, 7].

Dexmedetomidine, an alpha-2 adrenergic agonist, is increasingly favored for its sedative and analgesic effects, with less respiratory suppression and lower incidence of delirium compared to benzodiazepines [8, 9]. Meta-analyses indicate that Dexmedetomidine achieves satisfactory sedation in pediatric patients undergoing day care procedures [10]. Studies show comparable sedation effects with Dexmedetomidine for mechanically ventilated adults versus Midazolam and Propofol, along with benefits in delirium prevention, reduced mechanical ventilation duration, and shorter ICU stays [11–15]. In pediatric populations, Dexmedetomidine may provide better control over sedation in mechanical ventilation, especially postoperatively, though some studies, such as one involving an Indian cohort, have questioned its benefits in mechanically ventilated children [16–20].

Despite its advantages, Dexmedetomidine can cause hemodynamic side effects like hypotension and bradycardia, which necessitate close monitoring [17, 21]. Further large-scale controlled studies are needed to clarify its role in sedation for mechanically ventilated children. The present study aims to compare the efficacy and safety of Dexmedetomidine versus Midazolam in Iranian pediatric patients undergoing mechanical ventilation.

## Material and methods

### Study population and randomization

During February 2023 through February 2024 this randomized research was conducted across the three hospitals affiliated with Mashhad University of Medical sciences. This study received ethical approval from the Research Ethics Committee of Mashhad University of Medical Sciences while holding its registration at IRCT20190522043672N3 on 2023/2/16. Informed consent was obtained from the parents or legal guardians of all participants under the age of 16, and this has been clarified in the “Ethics approval and consent to participate” section of the manuscript.

### Inclusion criteria

Children aged 1 month to 18 years who required mechanical ventilation following rapid sequence endotracheal intubation (RSI), admitted to the PICU or emergency department, and whose parents or legal guardians provided written informed consent.

### Exclusion criteria

Patients with a known history of drug allergy or drug abuse, severe hepatic dysfunction (INR > 1.5), severe renal dysfunction (requiring dialysis), decreased level of consciousness or CNS disorders (encephalitis, status epilepticus, or other brain injuries), those receiving other sedatives during the study period, or who received a bolus sedative dose during mechanical ventilation, and patients with resistant hypotension or bradycardia.

All eligible participants were distributed between the Dexmedetomidine and the Midazolam intervention groups through a 1:1 random allocation method with generate a random sequence from http://www.graphpad.com/quickcalcs/index. Randomization and allocation were managed by a researcher who had no direct contact with the participants, ensuring blinding for both patients and other researchers. Blinding of treatment allocation was maintained for patients and researcher recording outcomes from the beginning to the end of the study, but the physician and nurse were aware of the medication prescribed to the patients.

### Sedation and analgesia

Researchers selected patients who received RSI and mechanical ventilation based on the inclusion criteria. Including only patients who underwent RSI allowed us to create a homogeneous study population, in which all subjects were intubated under controlled conditions before randomization and the start of continuous sedation. All patients were admitted to PICU and received continuous assessment through cardiac monitoring, noninvasive arterial blood pressure measurements and pulse oximetry. The Ramsay Sedation Scale was used to assess the level of consciousness and the target sedation score was set at 5 or 6. Its validity and reliability have been well documented by David Lozano-Díaz in 2021 [22].

During the rapid sequencing intubation, a bolus of fentanyl administered as an analgesic at the dose of 1 mcg/kg over 5 min, as well as sedative boluses consisted of Dexmedetomidine at 0.5—1 mcg/kg doses or Midazolam at 0.1- 0.2 mg/kg doses given over 5 min. Subsequently, along with the initiation of mechanical ventilation the continuous infusion of the drugs was immediately started as follows:

All children received intravenous infusion of Fentanyl at a standard dosage rate of 4 mcg/kg/h delivered through dedicated infusion pump.

- Dexmedetomidine was prescribed as a sedative for one group: the initial dose of Dexmedetomidine was 0.25 mcg/kg/h, infused for 15 min and then the researcher assessed patient's Ramsey score. If the score was less than 5, the drug was adjusted so that titrated by 0.25 mcg/kg/h every 15 min up to a maximum dose of 1 mcg/kg/h to achieve the target sedation score (5 0r 6).
- Midazolam was prescribed as a sedative for the other group: the initial dose of Midazolam was 1 mcg/kg/min, infused for 15 min and then the researcher assessed patient's Ramsey score. If the score was less than 5, the drug was adjusted so that titrated by 1 mcg/kg/min every 15 min up to a maximum dose of 4 mcg/kg/min to achieve the target sedation score (5 0r 6).

When maximum prescribed medication dosage proved insufficient for achieving target Ramsay score the medical team administered an additional sedative medication for patient as a rescue therapy while designating the situation as failed treatment. Treatment failure was defined as lack of achieving the target score even after administering the maximum specified dose of medication.

The patients meeting their sedation targets using drug doses within the prescription range were considered successful cases. The persistence of sedative effects of administered drugs were investigated by following up successful patients and reevaluation of their level of consciousness at 6 h after reaching target score during mechanical ventilation with three observed conditions.

- The same primary dose administered during start of ventilation phase helped maintain sedation at the set target level.
- Dosing adjustments for sedative drugs became necessary to preserve target sedation depths.
- The target Ramsay score remained unattainable even after reaching the drug's maximum dose allowance

The researcher documented Ramsay scores and vital signs including heart rate and mean arterial blood pressure at three different times during the study for the successful individuals: before the start of drug administration at baseline (T0), time to establish target sedation during the beginning of mechanical ventilation (T1) and 6 h after reaching target score (T2).

### Outcome variables

The primary outcome was the difference of success rates in achieving the target sedation level between the two groups among all patients aged one month to 18 years and young infants under one year of age.

Secondary outcomes were follow-up assessment of drug effectiveness at 6 h after reaching target score (T2) which was provided in three defined modes, effective dose of Dexmedetomidine and Midazolam to achieve target sedation levels during the start of mechanical ventilation (T1), hemodynamic complications throughout the treatment course including hypotension and bradycardia as safety indicators; hypotension was defined MAP < 55 mm Hg for infants below one year of age and MAP < 65 mm Hg for children above this age bracket and bradycardia was defined as heart rate < 60 for all ages, comparison of hemodynamic changes (MAP and HR) and Ramsay scores changes in successful patients between two groups at three time points (T0, T1, T3), duration of mechanical ventilation, duration of admission in PICU, Transition from controlled to assisted ventilation and mortality rate.

### Statistical analysis

The sample size was determined based on a previous study [23]. The calculation employed a formula for proportions using an inequality (offset) for two independent groups (unconditional), with a significance level (α) of 0.05 and a power of 90%. The estimated proportion of satisfactory sedation was 0.74 in the Dexmedetomidine group compared to 0.40 in the control group. Consequently, a minimum of 42 patients was required for each group. Considering potential dropouts, the final sample size was set to 60 individuals in each group (as illustrated in the CONSORT diagram).

Statistical analysis was performed using SPSS version 26.0. Quantitative data are presented as mean ± standard deviation and Median (IQR), while qualitative data are reported in percentages. Mann–Whitney U test was used to compare continuous variables between the two groups, and the χ^2^ test and Fisher’s exact test were employed for categorical variables and analysis of ANOVA with repeated measures performed to evaluate the average of MAP and HR at three consecutive measurements (T0, T1, T2). A p-value of < 0.05 was considered indicative of a statistically significant difference.

## Results

**Fig. 1:**
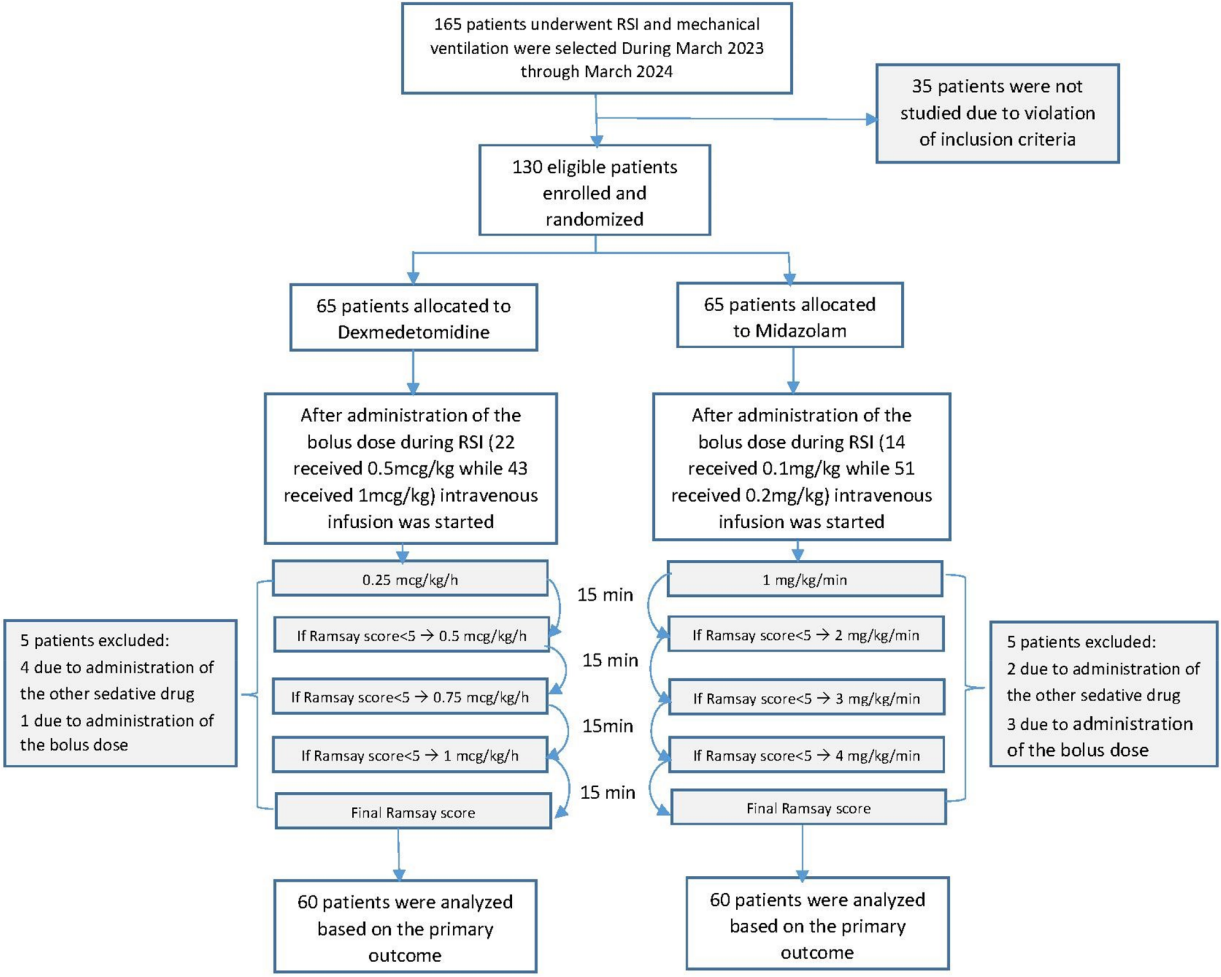
Algorithm of enrollment in the study

A total of 130 pediatric patients who met the inclusion criteria were enrolled during the study period from February 2023 to February 2024. As illustrated in Fig. 1 (CONSORT flow diagram), 65 patients were allocated to the dexmedetomidine group and 65 to the midazolam group. Five patients from each group were excluded due to the administration of additional sedative agents or bolus doses during mechanical ventilation. Consequently, 120 patients (60 in each group) completed the study and were included in the final analysis Fig. 1. Both groups in our study exhibited similar demographic characteristics, including age, gender, weight, and cause of hospitalization (pulmonary/non-pulmonary diseases) (Table 1).

**Table 1:** Baseline Characteristics of Mechanically Ventilated Children Enrolled in the Study

Characteristics		Dexmedetomidine (n = 60)	Midazolam (n = 60)	P value
Age (month), mean ± SD Median (IQR)		41.33 ± 44.88 22.5)12.0—66.75(	41.79 ± 50.32 20.0(11.75—62.0)	0.939*
Weight (kg), mean ± SD Median (IQR)		12.44 ± 7.83 10.0(8.0—18.5)	13.18 ± 11.00 10.0(7.0—14.0)	0.927*
sex	Male, n (%)	36 (60.0)	42 (70.0)	0.251**
	Female, n (%)	24 (40.0)	18 (30.0)	
Admission diagnosis	Pulmonary, n (%)	19 (31.7)	19 (31.7)	1.000**
	Non-pulmonary, n (%)	41 (68.3)	41 (68.3)	
Underlying disease	Cardiac, n (%)	5 (8.3)	4 (6.7)	0.879**
	Pulmonary, n (%)	2 (3.3)	3 (5.0)	
	Other, n (%)	21 (35.0)	18 (30.0)	
	Without disease, n (%)	32 (53.3)	35 (58.3)	

^*^ Mann–Whitney U; **Chi-squared test

Primary outcome: In children aged 1 month to 18 years, the success rate in achieving target sedation was significantly lower in the Dexmedetomidine group compared with Midazolam (p < 0.05). But in infants under 1 year, no significant difference was observed between groups (p = 0.157) (Table 2).

**Table 2:** Comparison of success rates in achieving target sedation at T1 (primary outcome)

variable		Dexmedetomidine	Midazolam	*P* value
**total patients**	**Success, n (%)**	38 (46.7)	54 (90.0)	< 0.001*
	**Failure, n (%)**	32 (53.3)	6 (10.0)	
	**Total, n (%)**	60	60	
**Infants under one year old**	**Success, n (%)**	20 (90.9)	21 (100.0)	0.157**
	**Failure, n (%)**	2 (9.1)	0 (0.0)	
	**Total, n (%)**	22 (100.0)	21 (100.0)	

^*^ Chi-squared test; **Fisher’s exact test

Baseline Ramsay scores were similar in two groups (p = 0.645 for 1 month-18 years; p = 0.978 for < 1 year). At the start of mechanical ventilation (T1), the mean final Ramsay score was significantly lower in the Dexmedetomidine group among children aged 1 month-18 years (p < 0.001; mean difference = 1.20) but not different in infants under 1 year (p = 0.083). Re-evaluation at 6 h (T2) showed that Dexmedetomidine-treated patients had lower Ramsay scores than the Midazolam group in both age ranges (p < 0.001; mean difference = 1.07 for 1 month–18 years, 1.24 for < 1 year) (Table 3).

**Table 3:** Comparison of Ramsay scores at three time points (T0, T1, T2)

Ramsay score	Dexmedetomidine (mean ± SD) Median (IQR)	Midazolam (mean ± SD) Median (IQR)	95% CI	Mean difference	P value*
Total patients					
Before sedation (T0)	1.66 ± 0.54 2.00 (1.00—2.00)	1.61 ± 0.64 2.00 (1.00—3.00)	−0.16, 0.25	0.05	0.645
Final score (T1)	4.40 ± 1.49 5.00 (3.00—6.00)	5.6 ± 0.80 6.00 (5.00—6.00)	−1.63, −0.76	−1.20	< 0.001
6 h later (T2)	4.57 ± 1.37 5.00 (3.00—6.00)	5.6 ± 0.75 6.00 (5.00—6.00)	−1.54, −0.60	−1.07	< 0.001
Infants under one year old					
Before sedation (T0)	1.47 ± 0.51 1.00 (1.00—2.00)	1.50 ± 0.60 1.00 (1.00—3.00)	−0.32, 0.28	−0.02	0.978
Final score (T1)	5.73 ± 0.61 6.00 (5.00—6.00)	5.86 ± 0.42 6.00 (5.00—6.00)	−0.39, 0.14	−0.12	0.083
6 h later (T2)	4.66 ± 1.39 5.00 (3.00—6.00)	5.91 ± 0.28 6.00 (5.00—6.00)	−1.73, −0.76	−1.24	< 0.001

Data are presented as mean ± standard deviation and Median (IQR); * Mann–Whitney

Three follow-up statuses were defined for successful patients. Follow-up analysis demonstrated a higher failure rate in the Dexmedetomidine group (35.7%) compared with Midazolam (7.4%, p < 0.001). About patients who maintained sedation, 46.5% of Dexmedetomidine-treated children required higher doses after 6 h, whereas 50% of Midazolam-treated patients remained stable on the initial dose. Dexmedetomidine was associated with fewer hemodynamic adverse effects, including hypotension (3.3% vs 23.3%) and bradycardia (p < 0.01). Change of ventilation mode, Mechanical ventilation duration and PICU stay were similar between groups (P = 0.640, P = 0.250 and p = 0.155), and no significant difference in mortality was observed (40% vs 41.7%, P = 0.853). (Table 4).

**Table 4:** Comparison of secondary outcomes

variable		Dexmedetomidine (n = 60)	Midazolam (n = 60)	P value
follow-up modes	Same initial dose, n (%)	5 (17.9)	27 (50)	< 0.001*
	Increased dose, n (%)	13 (46.4)	23 (42.6)	
	Failure, n (%)	10 (35.7)	4 (7.4)	
Hemodynamic complication	Age-adjusted hypotension, n (%)	2 (3.3)	14 (23.3)	< 0.01*
	Bradycardia (HR < 60), n (%)	0 (0.0)	0 (0.0)	
	Both, n (%)	0 (0.0)	1 (1.7)	
	None, n (%)	56 (96.7)	45 (75.0)	
Duration of intubation (day), mean ± SD Median (IQR)		8.70 ± 8.23 6.00 (3.00—9.00)	6.60 ± 4.58 5.50 (3.00—8.00)	0.250**
PICU stay (day), mean ± SD Median (IQR)		12.26 ± 8.23 10.00 (5.60—14.40)	10 ± 5.56 8.00 (4.50—11.50)	0.155**
Change of ventilatory mode, n (%)		8 (13.3%)	10 (16.7%)	0.640**
Mortality, n (%)		24 (40.0%)	25 (41.7)	0.853**

^*^ Chi-squared test; ** Mann–Whitney U

**Fig. 2:**
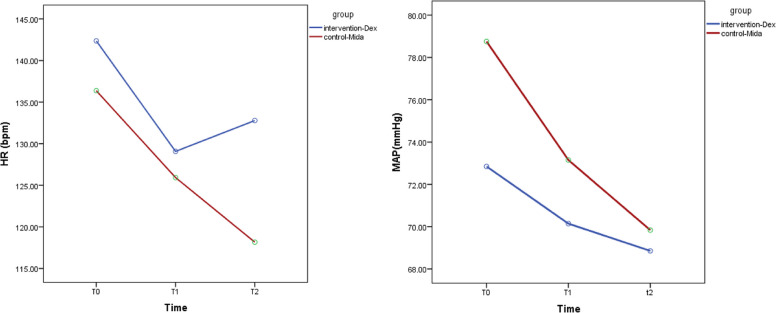
Comparison of the average of MAP and HR changes between the two groups

MAP and HR changes over T0, T1, and T2 were significant within each group (P = 0.035; P = 0.01) but did not differ between groups (P = 0.255 and 0.063, Fig. 2). Effective dose analysis showed that most patients achieved target sedation with 0.5 µg/kg/h of Dexmedetomidine, compared to 2 µg/kg/min of Midazolam (Table 5).

**Table 5:** Effective dose of sedative drugs on the first phase of mechanical ventilation (T1)

Dose	Dexmedetomidine (n = 60)	Midazolam (n = 60)
A, n (%)	7 (11.7)	16 (26.7)
B, n (%)	14 (23.3)	30 (50.0)
C, n (%)	3 (5.0)	4 (6.7)
D, n (%)	4 (6.7)	4 (6.7)
Rescue therapy, n (%)	32 (53.3)	6 (10.0)

Dexmedetomidine: (A = 0.25 mcg/kg/h; B = 0.5 mcg/kg/h; C = 0.75 mcg/kg/h; D = 1 mcg/kg/h)

Midazolam: (A = 1 mcg/kg/min; B = 2 mcg/kg/min; C = 3 mcg/kg/min; D = 4 mcg/kg/min)

## Discussion

In this randomized interventional trial, we evaluated the effectiveness and safety of Midazolam and Dexmedetomidine as sedative treatments for children on mechanical ventilation. Our study demonstrated that sedative and analgesic medications are crucial for mechanically ventilated children, improving ventilation efficacy and ensuring comfort [24]. Dexmedetomidine, a selective alpha-2 adrenergic agonist, provides sedative and analgesic effects with a rapid onset and offset of action, making it ideal for critical care settings without causing significant respiratory depression [25–27]. We excluded postoperative patients with elective intubation from our study, focusing on those who required urgent intubation for respiratory failure using RSI [28, 29].

The primary outcome of our study indicated that Dexmedetomidine was less effective than Midazolam in sedating pediatric patients aged one month to 18 years. Specifically, Dexmedetomidine could not replace Midazolam as an effective sedative for this age range. This finding is consistent with a recent study conducted in India, which also found that Dexmedetomidine did not demonstrate non-inferiority compared to Midazolam for sedation in mechanically ventilated children aged 1 month to 15 years. In that study, both groups had similar durations of mechanical ventilation, ICU stay, and hospital stay [20]. Conversely, other studies involving patients under 18 years suggest comparable efficacy between Dexmedetomidine and conventional sedative agents, such as Midazolam, particularly in post-surgical patients, a population difference that distinguishes these studies from ours [18, 19].

Interestingly, our research showed that Dexmedetomidine is as effective as Midazolam for infants under one year of age. A prospective observational cohort study evaluated sedation outcomes in 115 mechanically ventilated children under 13 year, with a median age of 12 months, demonstrating similar sedation durations at targeted levels set on 3–4 Ramsay score for Midazolam and Dexmedetomidine [3]. However, for the broader age group of one month to 18 years, Dexmedetomidine did not achieve the depth of sedation needed, as evidenced by the final Ramsey scores. The other research suggests that while Dexmedetomidine can induce light sedation and reduce Midazolam requirements, it may not provide deep sedation in older pediatric patients [30].

In infants under one year, Dexmedetomidine provided sedation equivalent to Midazolam at the start of mechanical ventilation. However, during the 6-h follow-up, most Dexmedetomidine-treated patients required higher doses to maintain target sedation, whereas about half of the Midazolam-treated patients retained stable sedation with their initial dose. Regarding safety, Dexmedetomidine was associated with fewer hemodynamic adverse effects, including age-adjusted hypotension and bradycardia, while all complications in both groups were manageable. Evidence from adult RCTs also supports that Dexmedetomidine may reduce delirium risk, mechanical ventilation duration, and ICU stay, although it may increase the risk of bradycardia without affecting mortality or hospital stay [15]. A systematic review that evaluated 8 RCTs involving 387 patients investigated Dexmedetomidine compared to other sedatives in mechanically ventilated children. It found that Dexmedetomidine was similar to other drugs regarding ICU stay, duration of sedation, and the need for additional sedatives, though it was associated with increased risks of bradycardia and hypotension without a difference in delirium occurrence [31].

Previous research performed on pediatric population has demonstrated equivalent or increased risk of hemodynamic complications including bradycardia and hypotension for Dexmedetomidine compared to other sedative drugs such as Midazolam [3, 19–21, 30]. The discrepancy between the results of previous studies and our study in terms of the incidence of hemodynamic complications may be due to the failure of our analysis to identify specific causes of complications so these adverse effects might stem either from disease progression or drug side effects.

The evaluation of changes in average of MAP and HR data recorded at three consecutive time points (T0, T1, T2) demonstrated significant decreases in these parameters in each group during the treatment period. However, when comparing two groups together, although blood pressure and heart rate alterations showed larger reductions in the Midazolam-treated group versus the Dexmedetomidine group yet statistical differences remained not significant. Patients in both groups experienced similar results concerning length of PICU stay, duration of mechanical ventilation and mortality outcomes.

Strength of our research is that it is one of the limited randomized controlled trials for child sedation during mechanical ventilation, specifically in Iranian healthcare settings. Despite the multicenter design, the present research contains several limitations which demand additional investigation. First, the inability to study post-operative patients undergoing elective intubation and not comparing them with those who received urgent intubation due to respiratory distress. Second, the lack of prolonged patient follow-up extends beyond 6 h. Third, failure to differentiate the cause of hemodynamic complications between drug-induced or disease-related adverse effects. We also did not examine other associated issues such as delirium, as well as the cost–benefit results. Further studies are needed to evaluate the efficacy and safety of Dexmedetomidine in children undergoing mechanical ventilation.

## Conclusion

The sedation benefits of Dexmedetomidine are inferior to Midazolam for children receiving mechanical ventilation aged 1 month to 18 years old, therefore it is not considered a suitable alternative for monotherapy. However, Dexmedetomidine can provides deep and sufficient sedative effects when administered to mechanically ventilated infants under one year of age. The use of intravenous Dexmedetomidine infusion presents a lower risk of bradycardia and hypotension when compared to Midazolam treatment. Light sedative effect with minimal hemodynamic complication of Dexmedetomidine in older children may make it suitable for procedures requiring low-level sedation as well as to reduce the dose of other sedatives used in mechanically ventilated patients as an adjunct therapy.

### What is already known

The effectiveness of Dexmedetomidine for sedation in mechanically ventilated children varies among populations. However, it is a safe drug with acceptable and reversible hemodynamic side effects.

### What new information this study adds

While dexmedetomidine is not ideal for deep sedation in older children receiving mechanical ventilation, it is an effective alternative for infants below one year. It is a safe drug and is associated with fewer hemodynamic side effects compared to midazolam.

## Data Availability

The data supporting the findings of this study are accessible through the research deputy of Mashhad University of Medical Sciences; however, access is restricted due to the licensing agreement under which these data were utilized for this study. Data may be available from the corresponding author upon reasonable request and with the approval of the research deputy of Mashhad University of Medical Sciences.
